# Sexual dysfunction and mode of delivery in Chinese primiparous women: a systematic review and meta-analysis

**DOI:** 10.1186/s12884-017-1583-2

**Published:** 2017-12-06

**Authors:** Dazhi Fan, Song Li, Wen Wang, Guo Tian, Li Liu, Song Wu, Xiaoling Guo, Zhengping Liu

**Affiliations:** 1 Foshan Institute of Fetal Medicine, Southern Medical University Affiliated Maternal & Child Health Hospital of Foshan, Foshan, Guangdong 528000 China; 2 Department of Obstetrics, Southern Medical University Affiliated Maternal & Child Health Hospital of Foshan, Foshan, Guangdong 528000 China; 3 Department of Epidemiology and Biostatistics, School of Public Health, Anhui Medical University, Hefei, Anhui 230032 China; 4 Chaohu Hospital Affiliated Anhui Medical University, Chaohu, Anhui 238000 China; 5 The First Affiliated Hospital, College of Medicine, Zhejiang University, Hang Zhou, Zhejiang 310003 China; 6 School of Integrated Traditional and Western Medicine, Anhui University of Chinese Medicine, Hefei, Anhui 230038 China

**Keywords:** Cesarean delivery, Vaginal delivery, Sexual function, Meta-analysis

## Abstract

**Background:**

Up to now, there is controversy over the effect of delivery mode cesarean delivery and spontaneous vaginal delivery on sexual function. Therefore, we did a systematic review and meta-analysis in postpartum women to explore the mode of delivery, cesarean delivery, and spontaneous vaginal delivery and differences in postpartum sexual function (short- and long-term) in Chinese primiparous women.

**Methods:**

Comprehensive electronic searches of PubMed, EMBASE, Web of Science, Elsevier Science Direct, Cochrane Library, the Chinese Biological Medical Literature database and the Chinese National Knowledge Infrastructure database were conducted to identify any study in each database published to August 31, 2017. The primary outcome was the sexual satisfaction and the secondary outcomes were resumed intercourse and sexual pain in the postpartum.

**Results:**

We identified 10 studies with a total population of 2851 in the present meta-analysis. Five and six eligible articles were respectively included for sexual satisfaction in postpartum at 3- and 6 months. Compared with vaginal delivery group, two time points were all not found statistically significance (OR 1.53, 95%CI 0.93–2.49; OR 1.15, 95%CI 0.95–1.39, respectively) in cesarean and spontaneous vaginal delivery group; in resumed intercourse and sexual pain domains, they were all significantly, with an overall OR of 2.05 (95%CI 1.36–3.11) at 3 months, 1.50 (95%CI 1.04–2.16) at 6 months and 0.29 (95%CI 0.24, 0.36) at 3 months, 0.73 (95%CI 0.58, 0.93) at 6 months, respectively. With the passage of time, the gap was closing. Sensitivity analysis was indicated a good stability of the meta-analysis in each domain.

**Conclusions:**

In conclusion, this meta-analysis indicated that the mode of delivery, cesarean and spontaneous vaginal delivery did not affect postpartum sexual satisfaction (short- and long-term) and appeared to have minimal effect on the long-term resumed intercourse and sexual pain in Chinese primiparous women. Primiparous women should be more cautious to choose cesarean section in order to preserve sexual function.

**Electronic supplementary material:**

The online version of this article (10.1186/s12884-017-1583-2) contains supplementary material, which is available to authorized users.

## Background

There has been concern about progressively increasing rates of caesarean section in many parts of the world, particularly among developing countries such as China [1, 2]. The caesarean section rate was more than 40% in many Chinese hospitals, while in some cases, it was up to 80% [3], which was now higher than the upper limit of 15% recommended by the WHO’s guidelines [4]. Long-term consequences associated with caesarean sections had been reported in many previous studies, including pelvic floor disorders, reduced fertility, placental abnormalities, mental distress, and female sexual dysfunction [5–7].

Female sexual function was considered a social issue, and pregnancy and delivery are considered contributory factors. Available data suggested that postpartum female sexual dysfunction was common in many countries [8–10]. The prevalence among postpartum women was as high as 41% to 83% in the first 3 months after delivery [8] and 18–30% of postpartum women still complained experiencing sexual problems at 6 months after delivery [9]. Among all domains of sexual problems, pain during intercourse, sexual dissatisfaction and delay in resuming intercourse were the most prevalent sexual problems for women and were negatively associated with the quality of life after childbirth [11–14]. In a study by Declercq and colleagues [12], almost half (48%) of mothers accused experiencing a painful perineum over the first 2 months postpartum, and 2% reported the pain persisting for at least 6 months.

Currently, Chinese women may prefer birth by caesarean section to vaginal delivery because they think it was safer and free from pain and anxiety and also may increase postpartum sexual life quality [15]. Up to now, numerous reports had been engaged with the task of exploring female sexuality in postpartum in Chinese women [16–25]. However, there was controversy over the effect of delivery mode cesarean delivery and spontaneous vaginal delivery on sexual function. Several studies had revealed the association between sexual function and delivery mode [18, 22, 23]. In contrary, other studies demonstrated no association between mode of delivery and sexual function [20, 21, 24].

Therefore, we did a systematic review and meta-analysis in postpartum women to explore the mode of delivery, cesarean delivery, and spontaneous vaginal delivery and differences in postpartum sexual function (short- and long-term) in Chinese primiparous women.

## Methods

This meta-analysis was performed following the guidelines from the Preferred Reporting Items for Systematic reviews and Meta-Analysis (PRISMA) statement (Additional file 1: Table S1) [26].

### Literature search

Identification and selection of relevant studies from PubMed, EMBASE, Web of Science, Elsevier Science Direct, Cochrane Library, the Chinese Biological Medical Literature database (CBM) and the Chinese National Knowledge Infrastructure database (CNKI) were searched for articles concerning the postpartum sexual function in Chinese primiparous women with planned/unplanned cesarean or spontaneous vaginal delivery mode. The last search update was on August 31, 2017. Keywords used to identify relevant articles were “mode of delivery”, “cesarean delivery”, “vaginal delivery”, and “sexual”, “sexual function” or “sexual dysfunction”. We used MeSH terms including “Delivery, Obstetric” [Mesh], “Cesarean Section” [Mesh], and “Sexual Dysfunction, Physiological” [Mesh]. Relevant articles in the reference lists were identified to obtain additional published studies.

### Inclusion and exclusion criteria

Studies meeting the following inclusion criteria were considered for this meta-analysis: (I) Clinical trials and prospective or retrospective studies investigating the correlation of the sexual function with the mode of delivery, planned/unplanned cesarean and spontaneous vaginal delivery, not instrumental delivery, among Chinese primiparous women; (II) Findings providing sufficient information for the estimation of odds ratios and 95% confidence intervals; and (III) the publication was in English or Chinese. Only studies published in peer-reviewed journals were included, data from letters and meetings abstracts were not eligible. Studies were also rejected if they did not meet the inclusion criteria or if they reported duplicated or useless data.

### Data extraction

Two authors (DF and SL) independently screened and determined the relevant studies and extracted the relevant data from each study and subsequently assessed the data to estimate reliability. The following information was obtained from the studies: first author, publication year, number of participants, age at time of delivery (mean, median, range), mode of delivery (planned/unplanned cesarean delivery, and spontaneous vaginal delivery), and sexual function outcomes (sexual satisfaction, resumed intercourse and sexual pain) in the first 3 and 6 months postpartum. The primary outcome of interest in this review was the sexual satisfaction in postpartum. And the secondary outcomes were resumed intercourse and sexual pain in postpartum. Any discrepancies were settled through discussion until a consensus was reached. Using Cohen’s kappa statistic, the consistency coefficient was 0.94 in the data extraction.

### Risk of bias assessment

Two authors (GT and LL) independently assessed the risk of bias of the selected studies using the Strengthening the Reporting of Observational Studies in Epidemiology (STROBE) Statement, which consisted of a checklist of 22 items [27]. Each item was classified as “no” (high risk), “yes” (low risk), or “unclear” to facilitate assessing the risk of potential bias in the title and abstract, introduction, methods, results, discussion, and other information sections of articles [27]. In order to report the results in percentages, the total number of “no”, “yes”, or “unclear” was added and divided by the total number of items for each study and multiplied by 100 [28]. A consensus reviewer (SW) resolved any observed discrepancies. The consistency coefficient was 0.96 in the risk of bias assessment.

### Statistical analysis

The ORs and 95% CIs were combined to obtain the effective value. A heterogeneity test based on *I* ^2^ and Q statistics was performed. The heterogeneity of individual ORs was calculated using Cochran chi-square (χ^2^) tests and quantified with the *I* ^2^ statistic. *I* ^2^ less than 25% denoted low heterogeneity, a value from 25 to 50% indicated moderate heterogeneity, and a value greater than 50% indicated substantial heterogeneity [29]. Significant heterogeneity was determined at a *p* value less than 0.10. The random effects model was used when heterogeneity was observed between primary studies. And the fixed effects model was used for analysis when no heterogeneity was observed. The impact was considered statistically significant when the 95% CI did not overlap with 1. Sensitivity analyses were performed to assess the stability of the results. Due to the small number of the included studies, publication bias cannot be assessed in this study. Statistical significance was considered for a *p*-value of less than 0.05 for summary OR. All calculations were performed using Review Manager Version 5.3 (provided by The Cochrane Collaboration, available from www.cc-ims.net/revman) and STATA version 11.0 (Stata Corporation, College Station, TX, USA).

## Results

### Literature search

**Fig. 1:**
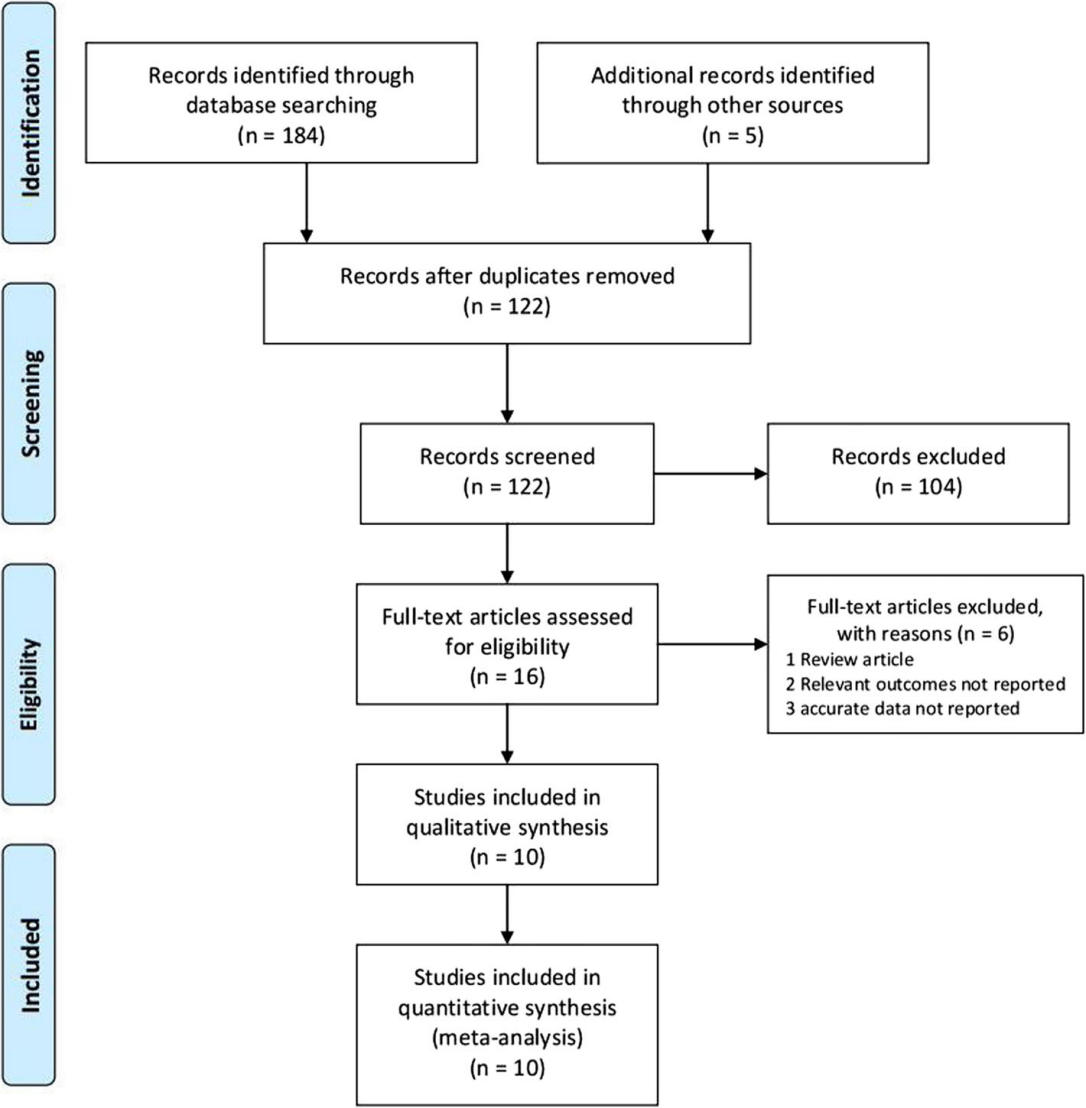
PRISMA flowchart showing the study selection process

**Fig. 2:**
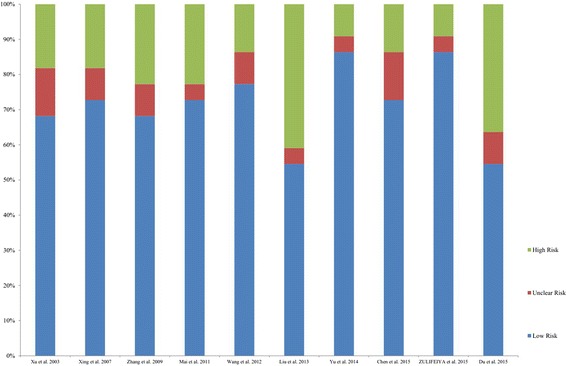
Risk of bias assessment using STROBE statement for each study**.** The statement consisted of a checklist of 22 items. Each item was classified as “no” (high risk), “yes” (low risk), or “unclear” to facilitate assessing the risk of potential bias in the title and abstract, introduction, methods, results, discussion, and other information sections of articles. In order to report the results in percentages, the total number of “no”, “yes”, or “unclear” was added and divided by the total number of items for each study and multiplied by 100

A total of 189 citations were retrieved from electronic databases. A flowchart of the study selection was provided in Fig. 1. After removal of duplicates, 122 publications were independently assessed for eligibility. After preliminary screening of titles and abstracts utilizing the aforementioned criteria, 16 articles were identified for full-text review. Of these, 6 were further excluded for various reasons (including 1 review article, 2 relevant outcomes not reported, and 3 accurate data not reported), leaving 10 eligible articles (2851 participants) [16–25] and were included in the meta-analysis. The study-level risk of bias results shown in Fig. 2. All of the included studies did not discuss limitations of the studies while nine studies did not give the source of funding and the role of the funders for the studies. Eight of ten studies did not describe any efforts to address potential sources of bias, explain how the study size was arrived at, or report other analyses done. The demographic characteristics of the participants in each study were shown in Table 1.

**Table 1:** Characteristics of individual studies included in meta-analysis

Author	Year	Number of participants		Age (years)^a^		Sexual Satisfaction		Resumed Intercourse		Sexual Pain	
		CD	VD	CD	VD	3 months	6 months	3 months	6 months	3 months	6 months
Xu XY	2003	260	142	–	–	N	Y	N	N	N	Y
Xing YX	2007	225	256	25.30 ± 2.15	24.6 ± 2.36	Y	Y	Y	Y	Y	Y
Zhang GP	2009	98	96	20–30	20–30	Y	Y	Y	Y	Y	Y
Mai XL	2011	206	224	25–29	25–29	N	N	Y	Y	Y	Y
Wang SG	2012	100	140	21–33	21–33	Y	Y	Y	Y	Y	Y
Liu D	2013	230	170	20–32	20–32	Y	Y	Y	Y	N	Y
Yu QY	2014	114	75	26.41 ± 2.25	26.39 ± 2.21	N	N	Y	Y	Y	N
Chen J	2014	100	100	25.90 ± 1.60	26.30 ± 2.10	Y	Y	Y	Y	Y	Y
ZULIFEIYA A	2015	115	80	25.50 ± 3.30	26.50 ± 3.50	N	N	Y	Y	N	N
Du ZL	2015	64	56	–	–	N	N	Y	N	Y	N

*CD* cesarean delivery, *VD* spontaneous vaginal delivery, *Y* yes, *N* no; −--, unknown

^a^Age was shown in mean ± standard deviation or minimum-maximum

### Sexual satisfaction

**Fig. 3:**
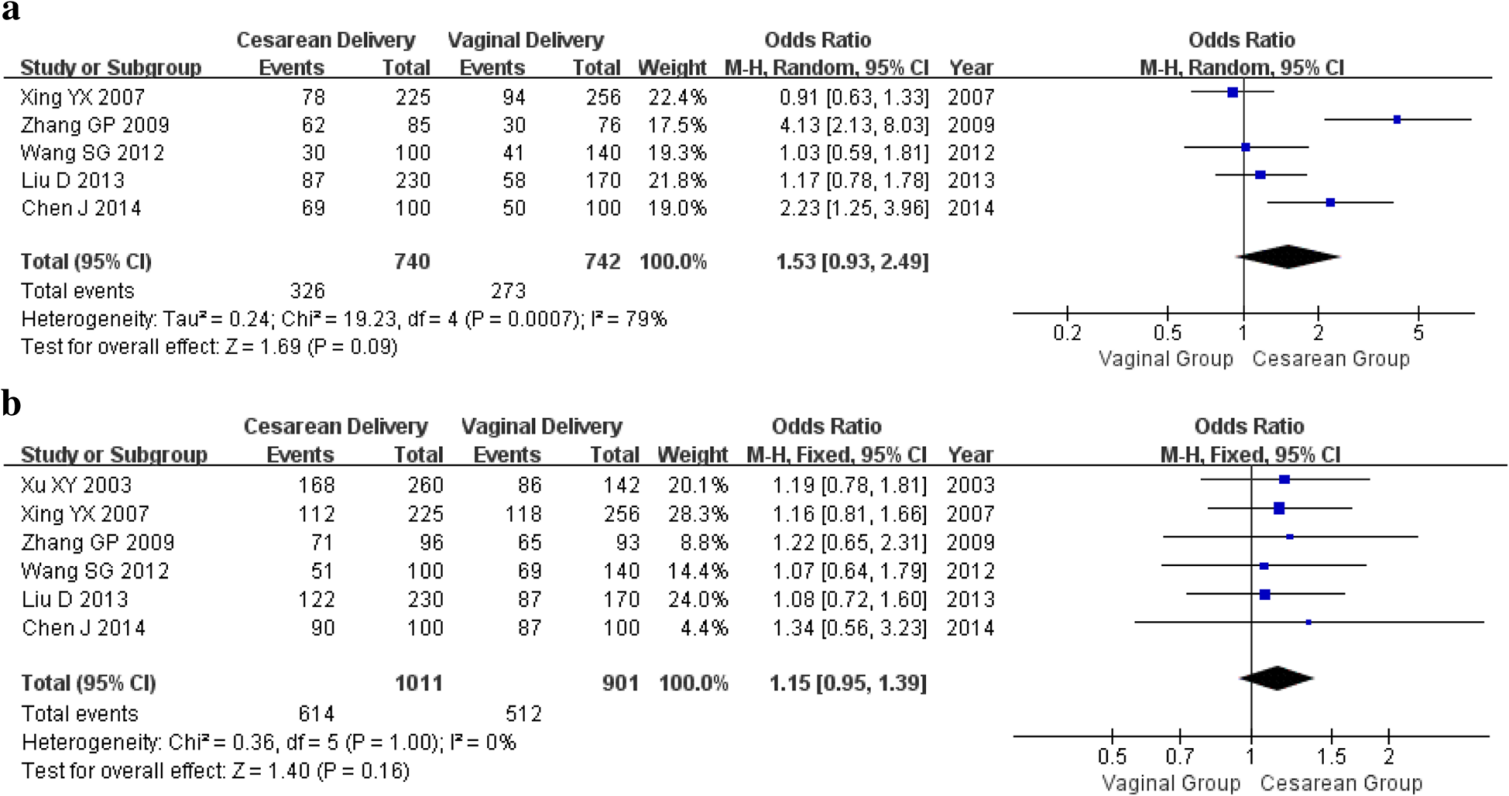
Forest plot of sexual satisfaction and mode of delivery**. a** within 3 months after delivery; (**b**) within 6 months after delivery

As showed in Table 2, 5 studies (740 participants in cesarean delivery group, 742 participants in spontaneous vaginal delivery group) were included in the meta-analysis for sexual satisfaction at 3 months postpartum. Six studies, including 1011 participants in cesarean delivery group and 901 participants in spontaneous vaginal delivery group, were included at 6 months postpartum. Compared with vaginal delivery group, there were all not found statistically significance in sexual satisfaction between the two groups at the two time points (OR 1.53, 95%CI 0.93–2.49; OR 1.15, 95%CI 0.95–1.39, respectively) (Fig. 3).

**Table 2:** Meta-analysis of the association between mode of delivery and postpartum sexual function

Variables		Eligible	Number		OR (95% CI)	*P*	Heterogeneity Test		Effect Model
		Studies	CD	VD			*P*	*I* ^2^ (%)	
Sexual Satisfaction									
	3 month	5	740	742	1.53 (0.93, 2.49)	0.09	0.0007	79	Random
	6 month	6	1011	901	1.15 (0.95, 1.39)	0.16	0.99	0	Fixed
Resumed Intercourse									
	3 month	9	1252	1197	2.05 (1.36, 3.11)	0.0007	<0.0001	77	Random
	6 month	8	1188	1141	1.50 (1.04, 2.16)	0.03	0.56	0	Fixed
Sexual Pain									
	3 month	7	1010	1022	0.29 (0.24, 0.36)	<0.00001	0.63	0	Fixed
	6 month	7	1223	1126	0.73 (0.58, 0.93)	0.01	0.76	0	Fixed

*CD* cesarean delivery, *VD* spontaneous vaginal delivery, *OR* odds ratios, *95%CI* 95% confidence interval

Sensitivity analysis was performed to assess the stability of the meta-analysis. When any single study was deleted, the corresponding pooled ORs were changed slightly (Additional file 2: Table S2; Additional file 3: Figures S1–S2), with the statistically similar results indicating a satisfactory stability of the meta-analysis.

### Resumed intercourse and sexual pain

**Fig. 4:**
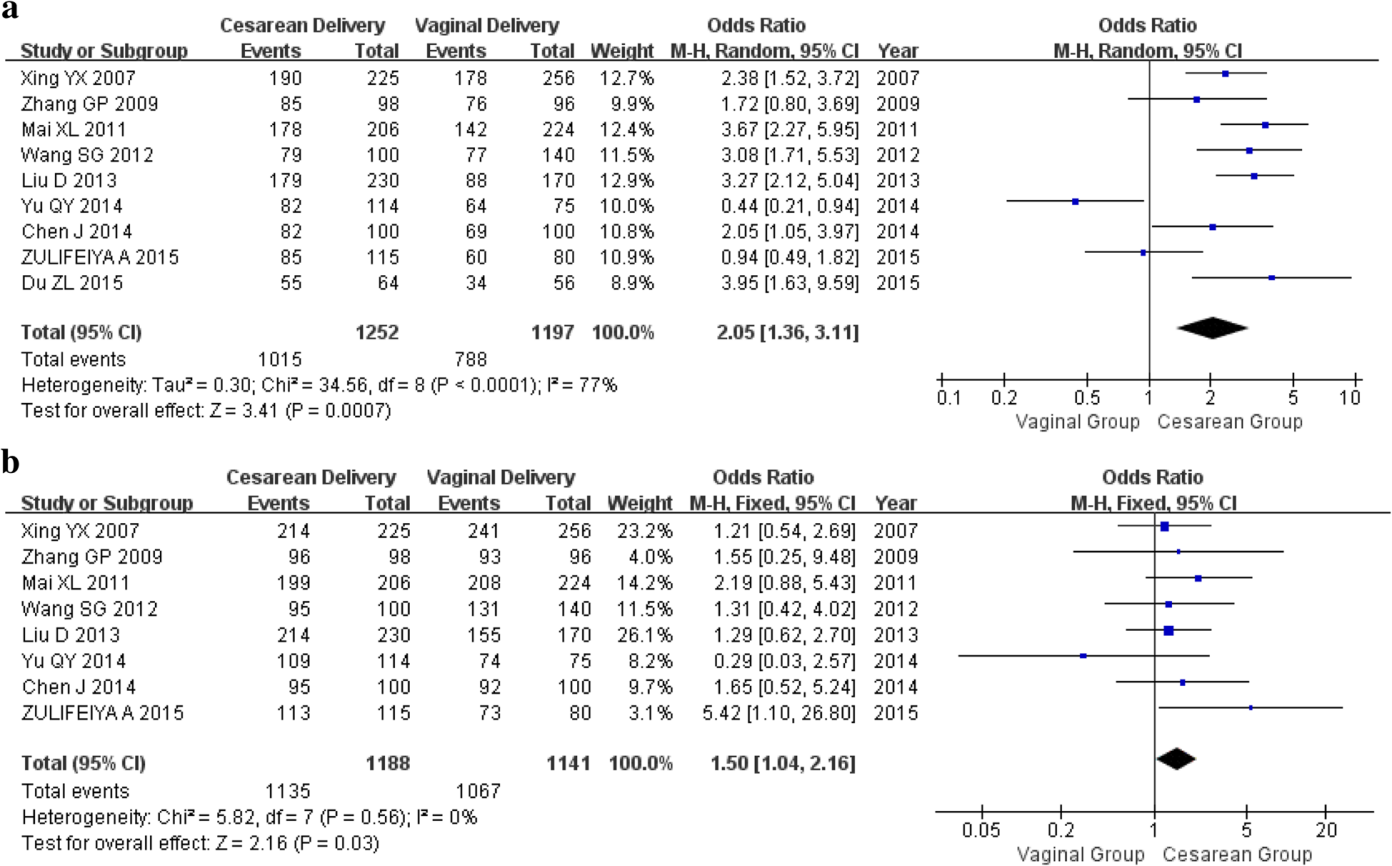
Forest plot of resumed intercourse and mode of delivery**. a** within 3 months after delivery; (**b**) within 6 months after delivery

Nine (2449 participants) and eight (2329 participants) studies were included for resumed intercourse in postpartum at 3 months and 6 months, respectively. Compared with vaginal delivery group, participants were all earlier resumed intercourse in cesarean delivery group at 3 months and 6 months postpartum, with an overall OR of 2.05 (95% CI 1.36–3.11) at 3 months in random model (*p* < 0.0001, *I* ^2^ = 77%) and OR of 1.50 (95% CI 1.04–2.16) at 6 months in fixed model (*p* < 0.56, *I* ^2^ = 0%), respectively (Fig. 4).

**Fig. 5:**
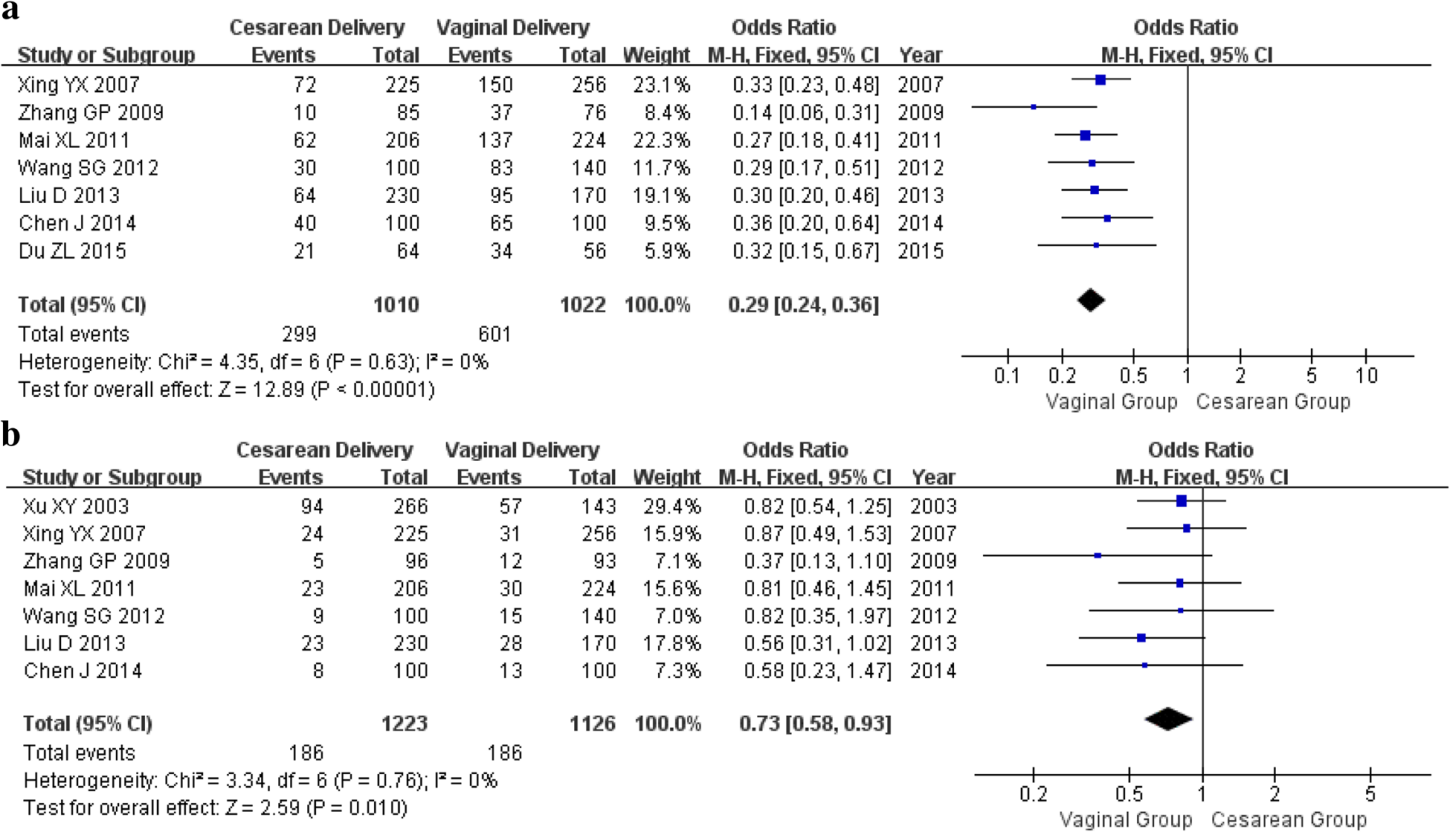
Forest plot of sexual pain and mode of delivery**. a** within 3 months after delivery; (**b**) within 6 months after delivery

In the sexual pain domain, there were 2032 (seven articles) and 2349 participants (seven articles) in postpartum at 3 months and 6 months, respectively. Compared with vaginal delivery group, less sexual pain was all observed in cesarean delivery group at 3 months (OR = 0.29, 95%CI 0.24, 0.36) and at 6 months (OR = 0.73, 95%CI 0.58, 0.93), respectively (Fig. 5). Because of the insignificant heterogeneity between studies, they were all determined using the fixed effect method (*p* < 0.63, *I* ^2^ = 0%, *p* < 0.76, *I* ^2^ = 0%; respectively).

When any single study was deleted, sensitivity analysis was indicated a good stability of the meta-analysis in each domain (Additional file 2: Table S2; Additional file 3: Figures S3–S6).

## Discussion

To the best of our knowledge, this is the first meta-analysis that examines the results association between postpartum sexual function and the mode of delivery, cesarean and spontaneous vaginal delivery, in Chinese primiparous women. The results indicated that two of the three domains (resumed intercourse and sexual pain) in both short-term (3 months later) and long-term (6 months later) were all better in cesarean delivery group. However, with the passage of time, the gap was closing. The sexual satisfaction was all not found different at short- and long-term postpartum in the two delivery models.

Sexual health is an important aspect of quality of life. Studies [30–32] have reported that diseases could affect the sexual function. Many studies have focused on the mode of delivery and postpartum sexual health [33–35], however, these results are still inconsistent. In a cross-sectional postal survey study [34], Dean and colleagues showed that sexual satisfaction was significantly less in women with vaginal delivery than the women who had undergone a cesarean section. However, in another similar cross-sectional study of primiparous women [35], Hosseini et al. reported that women in the normal vaginal delivery group and planned cesarean section group had no significant difference in the sexual function, including sexual satisfaction and pain, and he appealed that undergoing planned cesarean section in order to preserve sexual function was not recommended.

In this meta-analysis study, the results indicated that sexual satisfaction was all not found different in the 3 and 6 months postpartum. In the study of 1159 Canadian postpartum women [36], Hannah and colleagues reported that sexual satisfaction from 3 months to 2 years postpartum was similar among women who had undergone a planned cesarean section and planned vaginal birth. Meanwhile, similar results were also showed in other studies [34, 37, 38].

Although other two domains (resumed intercourse and sexual pain) in both short-term (3 months later) and long-term (6 months later) were better in cesarean delivery group, with the passage of time, the gap was closing. In the study by a postal survey conducted on 484 British primiparous women at 6 months after delivery [39], resumption of sexual intercourse did not differ significantly by type of birth. Meanwhile, women who had undergone cesarean sections were significantly less likely to experience sexual dysfunction at 3 months postpartum, but there was no significant difference at 6 months.

The results of the sensitivity analysis were not materially altered and did not draw different conclusions, indicating that the initial results were strong. However, some limitations also need to be discussed. First, this meta-analysis was based on data from studies whose results had been published, and some relevant studies could not be included in our analysis. Due to the small number of the included studies, publication bias cannot be assessed. Meanwhile, some items of the STROBE Statement were not reported in the included studies, which could increase the high risk of bias. Second, the meta-analysis was limited to different adjustments for potential confounding variables (age, area, primiparous/multiparous, et al.) in each study. Because of lacking sufficient data, we cannot exclude the possibility that these factors, which may affect the final conclusion of the present study. Third, the results were based on self-created questionnaire among studies, which may result in some misclassification bias. In addition, various outcome measures (resumption of sexual activity, satisfaction or orgasm, sexual sensation, sexual inactivity) assessed the postpartum sexual problems for women. Because most Chinese doctors just focus on the three domains, we cannot assess all of the domains of their postpartum sexual problems.

## Conclusions

This meta-analysis indicated that the mode of delivery, cesarean delivery, and vaginal delivery did not affect postpartum sexual satisfaction (short- and long-term) and appeared to have minimal effect on the long-term resumed intercourse and sexual pain in Chinese primiparous women. Postpartum sexual dysfunction may be affected to prepartum sexual function, the partner’s experience, and other social and cultural factors. Those requesting caesarean delivery without conventional medical indications or obstetric indications for foetus or mother, should be advised of these potential results.

It is important to inform pregnant women that the mode of delivery is not a major factor in postpartum sexual dysfunction. Meanwhile, in clinical practice, more attention should be given to the relationship between the mode of delivery and postpartum sexual function, especially primiparous women. Obstetricians, nursing staffs, and all of the health care providers who are engaged in counseling couples during the antepartum period should be told in order to train couples regarding potential postpartum sexual dysfunction to help these couples chose the mode of delivery.

## Additional files

Additional file 1: Table S1. PRISMA Checklist. (DOC 64 kb)

Additional file 2: Table S2. The results of the included studies through sensitivity analysis. (DOC 98 kb)

Additional file 3: The results of the included studies through sensitivity analysis. **Figure S1**. Sensitivity analysis of 5 studies with the random effects model for sexual satisfaction within 3 months after delivery. **Figure S2**. Sensitivity analysis of 6 studies with the fixed effects model for sexual satisfaction within 6 months after delivery. **Figure S3**. Sensitivity analysis of 9 studies with the random effects model for resumed intercourse within 3 months after delivery. **Figure S4**. Sensitivity analysis of 8 studies with the fixed effects model for resumed intercourse within 6 months after delivery. **Figure S5**. Sensitivity analysis of 7 studies with the fixed effects model for sexual pain within 3 months after delivery. **Figure S6**. Sensitivity analysis of 7 studies with the fixed effects model for sexual pain within 6 months after delivery. (DOCX 957 kb)

## Abbreviations

CBM: The Chinese biological medical literature database
CD: Cesarean delivery
CI: Confidence interval
CNKI: The Chinese national knowledge infrastructure database
NOS: Newcastle-Ottawa Scale
OR: Odds ratio
PRISMA: The preferred reporting items for systematic reviews and meta-analysis
STROBE: The strengthening the reporting of observational studies in epidemiology
VD: Spontaneous vaginal delivery
