# Supplementary material for: Sexual dysfunction and mode of delivery in Chinese primiparous women: a systematic review and meta-analysis
Source: BMC Pregnancy Childbirth. 2017 Dec 6;17:408. doi: 10.1186/s12884-017-1583-2 (PMC5719940; doi:10.1186/s12884-017-1583-2)
Supplement: Supplementary file 2 — The results of the included studies through sensitivity analysis. (DOC 98 kb) [file 12884_2017_1583_MOESM2_ESM.doc]

**S2** Table. The results of the included studies through sensitivity analysis

| Excluded study | CD (Number) | VD (Number) | OR (95% CI) | *P* value | Heterogeneity test | Effect model |
| --- | --- | --- | --- | --- | --- | --- |
| **Sexual Satisfaction** |  |  |  |  |  |  |
| 3 months |  |  |  |  |  |  |
| Before excluding | 740 | 742 | 1.53 (0.93, 2.49) | 0.09 | *P* =0.0007,*I^2^*=79% | Random |
| Xing YX 2007 | 515 | 486 | 1.77 (0.99, 3.17) | 0.05 | *P* =0.004,*I^2^*=78% | Random |
| Zhang GP 2009 | 655 | 666 | 1.21 (0.85, 1.71) | 0.29 | *P* =0.09,*I^2^*=55% | Random |
| Wang SG 2012 | 640 | 602 | 1.69 (0.92, 3.09) | 0.09 | *P* =0.0004,*I^2^*=84% | Random |
| Liu D 2013 | 510 | 572 | 1.66 (0.86, 3.23) | 0.13 | *P* =0.0003,*I^2^*=84% | Random |
| Chen J 2014 | 640 | 642 | 1.40 (0.80, 2.43) | 0.24 | *P* =0.001,*I^2^*=81% | Random |
| 6 months |  |  |  |  |  |  |
| Before excluding | 1011 | 901 | 1.15 (0.95, 1.39) | 0.16 | *P* =0.99,*I^2^*=0% | Fixed |
| Xu XY 2003 | 751 | 759 | 1.14 (0.92, 1.41) | 0.24 | *P* =0.99,*I^2^*=0% | Fixed |
| Xing YX 2007 | 786 | 645 | 1.14 (0.91, 1.43) | 0.25 | *P* =0.99,*I^2^*=0% | Fixed |
| Zhang GP 2009 | 915 | 808 | 1.14 (0.93, 1.39) | 0.20 | *P* =0.99,*I^2^*=0% | Fixed |
| Wang SG 2012 | 911 | 761 | 1.16 (0.94, 1.43) | 0.16 | *P* =0.99,*I^2^*=0% | Fixed |
| Liu D 2013 | 781 | 731 | 1.17 (0.94, 1.45) | 0.16 | *P* =0.99,*I^2^*=0% | Fixed |
| Chen J 2014 | 911 | 801 | 1.14 (0.94, 1.38) | 0.20 | *P* =0.99,*I^2^*=0% | Fixed |
| **Resumed Intercourse** |  |  |  |  |  |  |
| 3 months |  |  |  |  |  |  |
| Before excluding | 1252 | 1197 | 2.05 (1.36, 3.11) | 0.0007 | *P* =0.0001,*I^2^*=77% | Random |
| Xing YX 2007 | 1027 | 941 | 2.00 (1.23, 3.26) | 0.006 | *P* =0.0001,*I^2^*=80% | Random |
| Zhang GP 2009 | 1154 | 1101 | 2.09 (1.33, 3.29) | 0.001 | *P* =0.0001,*I^2^*=79% | Random |
| Mai XL 2011 | 1046 | 973 | 1.89 (1.20, 2.97) | 0.006 | *P* =0.0001,*I^2^*=77% | Random |
| Wang SG 2012 | 1152 | 1057 | 1.94 (1.22, 3.09) | 0.005 | *P* =0.0001,*I^2^*=79% | Random |
| Liu D 2013 | 1022 | 1027 | 1.91 (1.20, 3.06) | 0.007 | *P* =0.0001,*I^2^*=78% | Random |
| Yu QY 2014 | 1138 | 1122 | 2.47 (1.82, 3.53) | 0.00001 | *P* =0.03,*I^2^*=54% | Random |
| Chen J 2014 | 1152 | 1097 | 2.05 (1.29, 3.25) | 0.002 | *P* =0.0001,*I^2^*=80% | Random |
| Du ZL 2015 | 1188 | 1141 | 1.92 (1.24, 2.99) | 0.004 | *P* =0.0001,*I^2^*=79% | Random |
| ZULIFEIYA A 2015 | 1137 | 1117 | 2.27 (1.50, 3.42) | 0.0001 | *P* =0.0003,*I^2^*=74% | Random |
| 6 months |  |  |  |  |  |  |
| Before excluding | 1188 | 1141 | 1.50 (1.04, 2.16) | 0.03 | *P* =0.56,*I^2^*=0% | Fixed |
| Xing YX 2007 | 963 | 885 | 1.58 (1.05, 2.39) | 0.03 | *P* =0.48,*I^2^*=0% | Fixed |
| Zhang GP 2009 | 1090 | 1045 | 1.49 (1.03, 2.17) | 0.04 | *P* =0.44,*I^2^*=0% | Fixed |
| Mai XL 2011 | 982 | 917 | 1.38 (0.92, 2.06) | 0.11 | *P* =0.54,*I^2^*=0% | Fixed |
| Wang SG 2012 | 1088 | 1001 | 1.52 (1.03, 2.24) | 0.03 | *P* =0.45,*I^2^*=0% | Fixed |
| Liu D 2013 | 958 | 971 | 1.57 (1.03, 2.39) | 0.04 | *P* =0.47,*I^2^*=0% | Fixed |
| Yu QY 2014 | 1074 | 1066 | 1.60 (1.10, 2.34) | 0.01 | *P* =0.73,*I^2^*=0% | Fixed |
| Chen J 2014 | 1088 | 1041 | 1.48 (1.01, 2.17) | 0.05 | *P* =0.45,*I^2^*=0% | Fixed |
| ZULIFEIYA A 2015 | 1073 | 1061 | 1.37 (0.94, 2.00) | 0.11 | *P* =0.78,*I^2^*=0% | Fixed |
| **Sexual Pain** |  |  |  |  |  |  |
| 3 months |  |  |  |  |  |  |
| Before excluding | 1010 | 1022 | 0.29 (0.24, 0.36) | 0.0001 | *P* =0.63,*I^2^*=0% | Fixed |
| Xing YX 2007 | 785 | 766 | 0.28 (0.23, 0.35) | 0.0001 | *P* =0.57,*I^2^*=0% | Fixed |
| Zhang GP 2009 | 925 | 946 | 0.31 (0.26, 0.37) | 0.0001 | *P* =0.98,*I^2^*=0% | Fixed |
| Mai XL 2011 | 804 | 798 | 0.30 (0.24, 0.37) | 0.0001 | *P* =0.53,*I^2^*=0% | Fixed |
| Wang SG 2012 | 910 | 882 | 0.30 (0.24, 0.36) | 0.0001 | *P* =0.50,*I^2^*=0% | Fixed |
| Liu D 2013 | 780 | 852 | 0.29 (0.24, 0.36) | 0.0001 | *P* =0.50,*I^2^*=0% | Fixed |
| Chen J 2014 | 910 | 922 | 0.29 (0.24, 0.35) | 0.0001 | *P* =0.57,*I^2^*=0% | Fixed |
| Du ZL 2015 | 946 | 966 | 0.29 (0.24, 0.36) | 0.0001 | *P* =0.50,*I^2^*=0% | Fixed |
| 6 months |  |  |  |  |  |  |
| Before excluding | 1223 | 1126 | 0.73 (0.58, 0.93) | 0.01 | *P* =0.76,*I^2^*=0% | Fixed |
| Xu XY 2003 | 957 | 983 | 0.70 (0.52, 0.92) | 0.01 | *P* =0.71,*I^2^*=0% | Fixed |
| Xing YX 2007 | 998 | 870 | 0.71 (0.55, 0.92) | 0.009 | *P* =0.71,*I^2^*=0% | Fixed |
| Zhang GP 2009 | 1127 | 1033 | 0.76 (0.60, 0.97) | 0.03 | *P* =0.88,*I^2^*=0% | Fixed |
| Mai XL 2011 | 1017 | 902 | 0.72 (0.56, 0.93) | 0.01 | *P* =0.67,*I^2^*=0% | Fixed |
| Wang SG 2012 | 1123 | 986 | 0.73 (0.57, 0.93) | 0.01 | *P* =0.66,*I^2^*=0% | Fixed |
| Liu D 2013 | 993 | 956 | 0.77 (0.60, 0.99) | 0.05 | *P* =0.79,*I^2^*=0% | Fixed |
| Chen J 2014 | 1123 | 1026 | 0.75 (0.58, 0.95) | 0.02 | *P* =0.69,*I^2^*=0% | Fixed |

CD, cesarean delivery; VD, spontaneous vaginal delivery; OR, odds ratios; 95%CI, 95% confidence interval;
