# Supplementary material for: Sexual dysfunction and mode of delivery in Chinese primiparous women: a systematic review and meta-analysis
Source: BMC Pregnancy Childbirth. 2017 Dec 6;17:408. doi: 10.1186/s12884-017-1583-2 (PMC5719940; doi:10.1186/s12884-017-1583-2)
Supplement: Supplementary file 3 — The results of the included studies through sensitivity analysis. Figure S1. Sensitivity analysis of 5 studies with the random effects model for sexual satisfaction within 3 months after delivery. Figure S2. Sensitivity analysis of 6 studies with the fixed effects model for sexual satisfaction within 6 months after delivery. Figure S3. Sensitivity analysis of 9 studies with the random effects model for resumed intercourse within 3 months after delivery. Figure S4. Sensitivity analysis of 8 studies with the fixed effects model for resumed intercourse within 6 months after delivery. Figure S5. Sensitivity analysis of 7 studies with the fixed effects model for sexual pain within 3 months after delivery. Figure S6. Sensitivity analysis of 7 studies with the fixed effects model for sexual pain within 6 months after delivery. (DOCX 957 kb) [file 12884_2017_1583_MOESM3_ESM.docx]

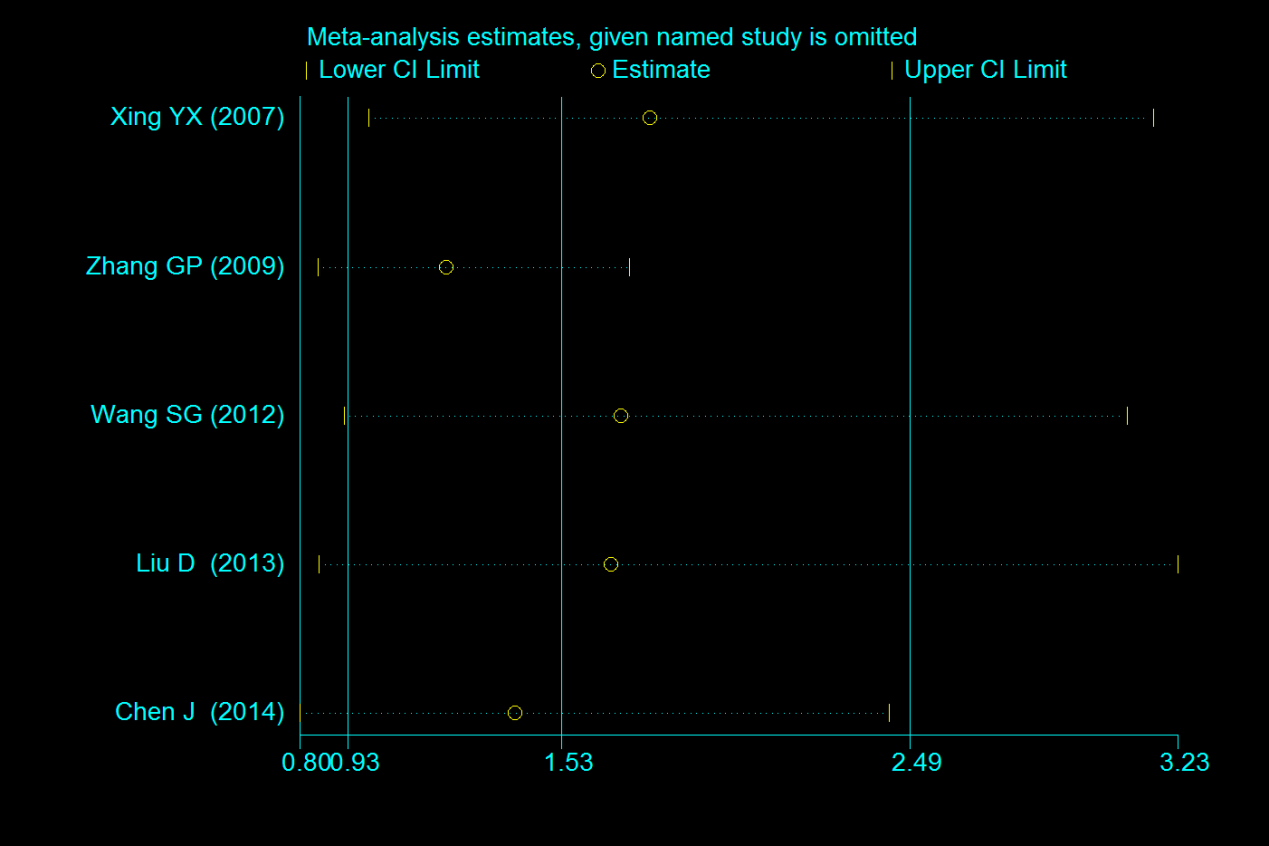


**S1 Fig. Sensitivity analysis of 5 studies with the random effects model for sexual satisfaction within 3 months after delivery.**


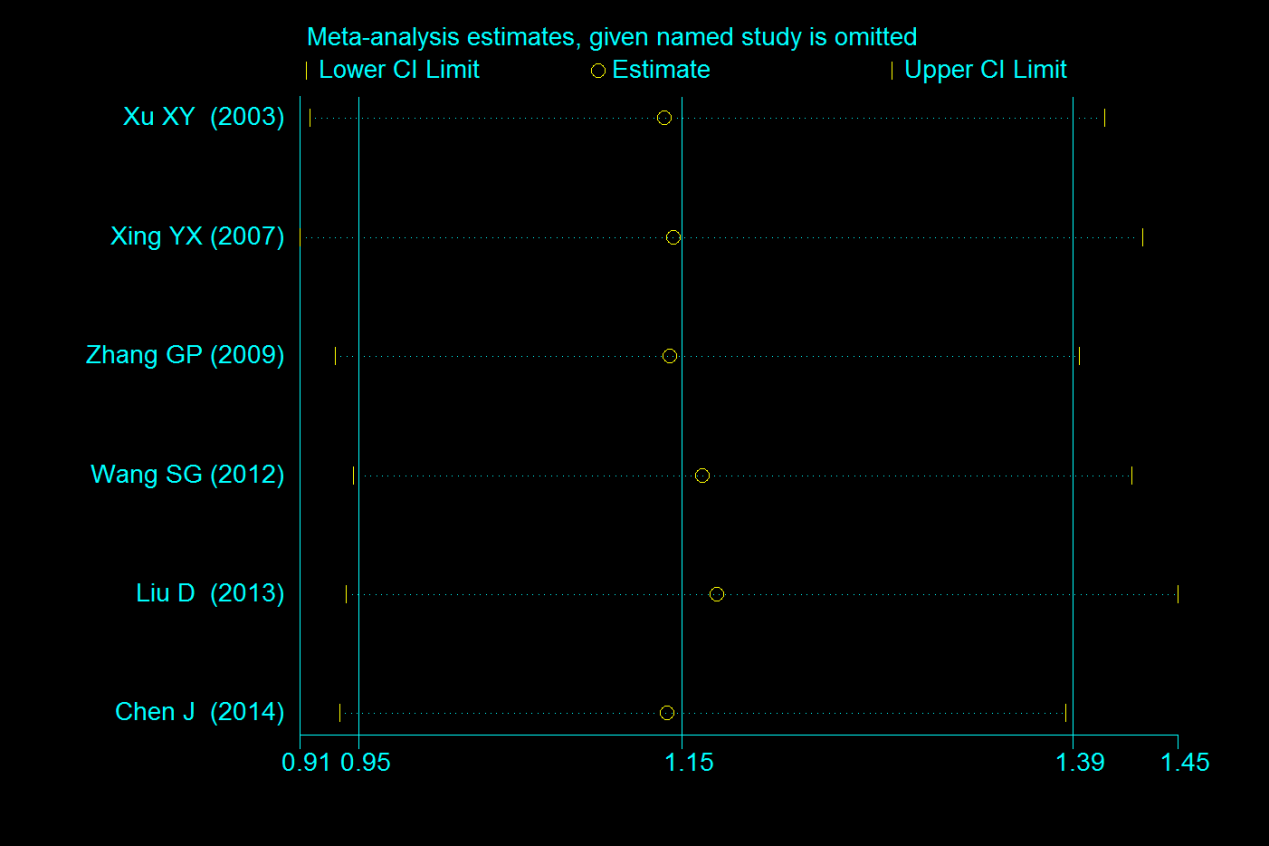


**S2 Fig. Sensitivity analysis of 6 studies with the fixed effects model for sexual satisfaction within 6 months after delivery.**


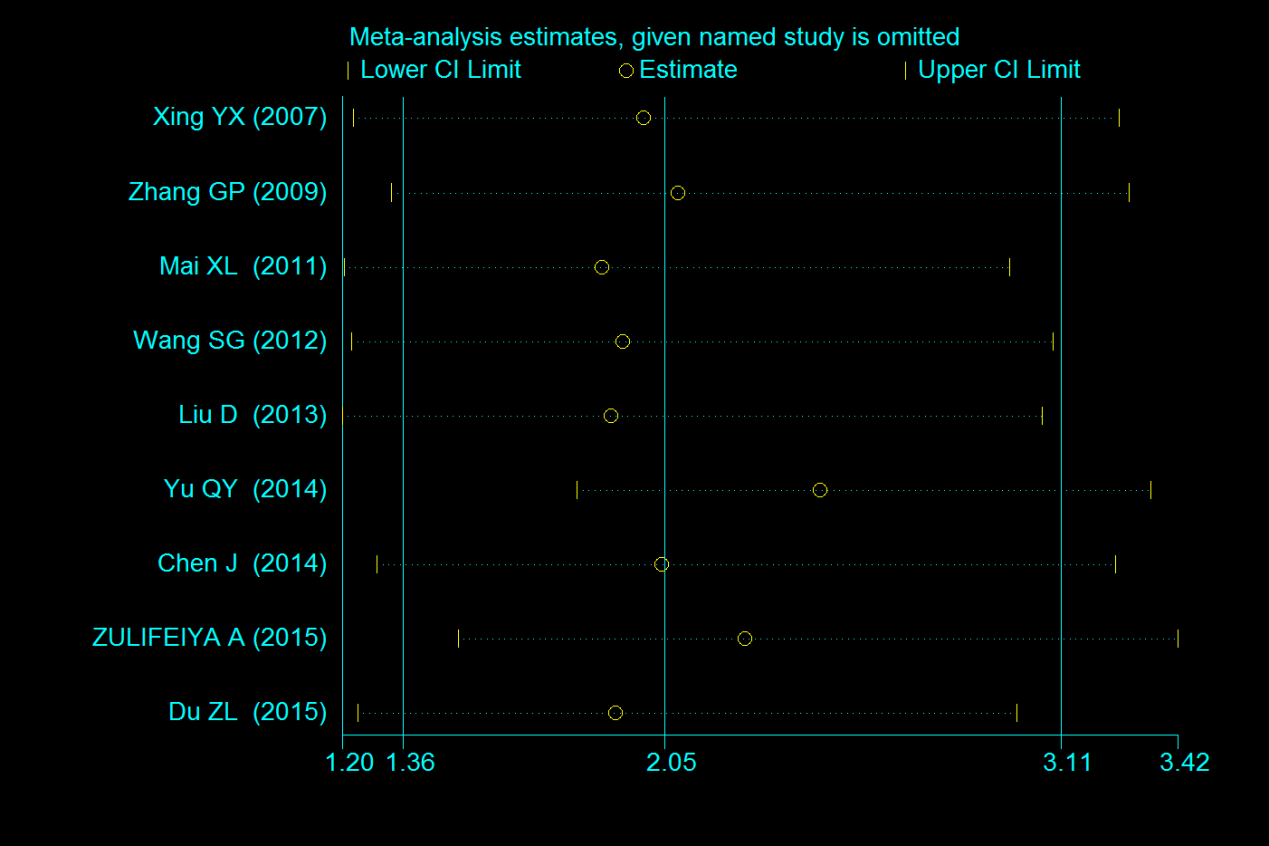


**S3 Fig. Sensitivity analysis of 9 studies with the random effects model for resumed intercourse within 3 months after delivery.**


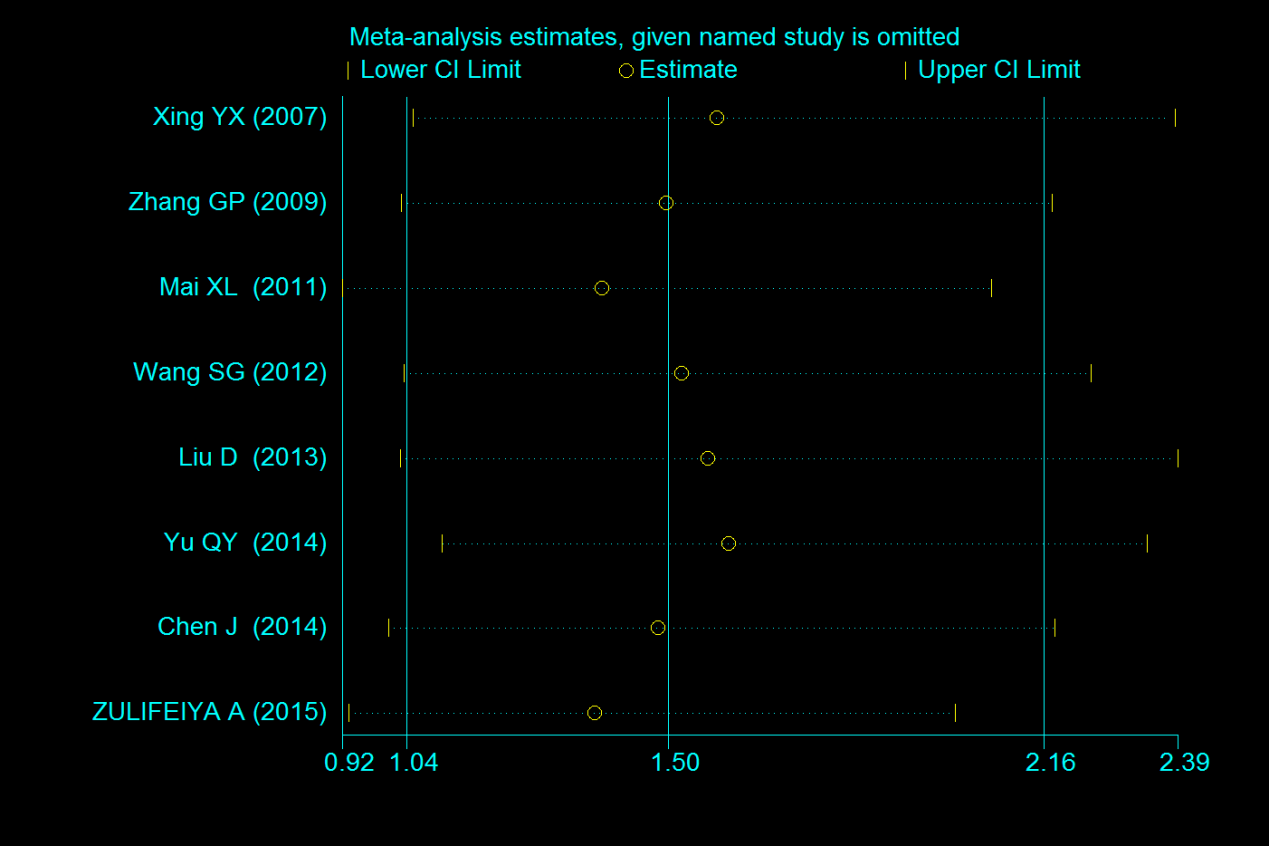


**S4 Fig. Sensitivity analysis of 8 studies with the fixed effects model for resumed intercourse within 6 months after delivery.**


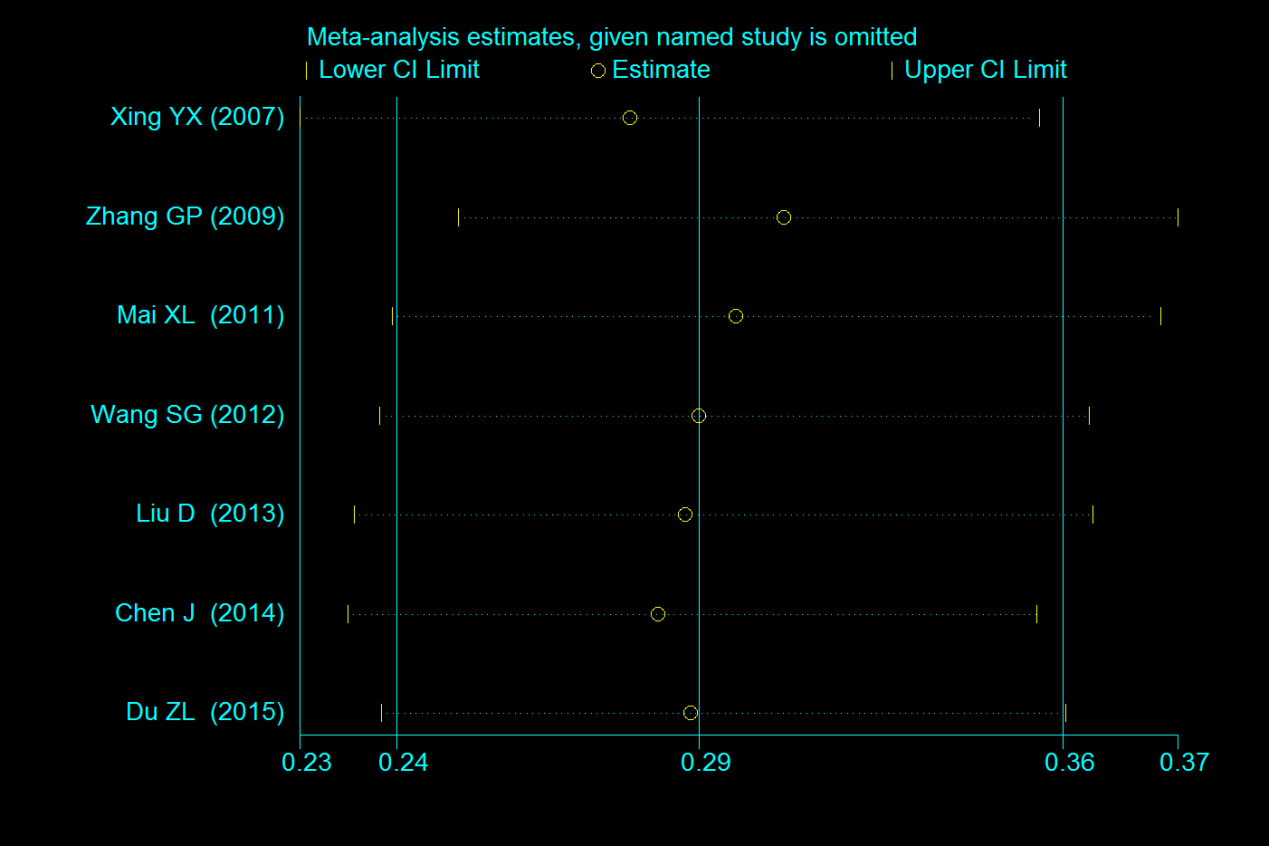


**S5 Fig. Sensitivity analysis of 7 studies with the fixed effects model for sexual pain within 3 months after delivery.**


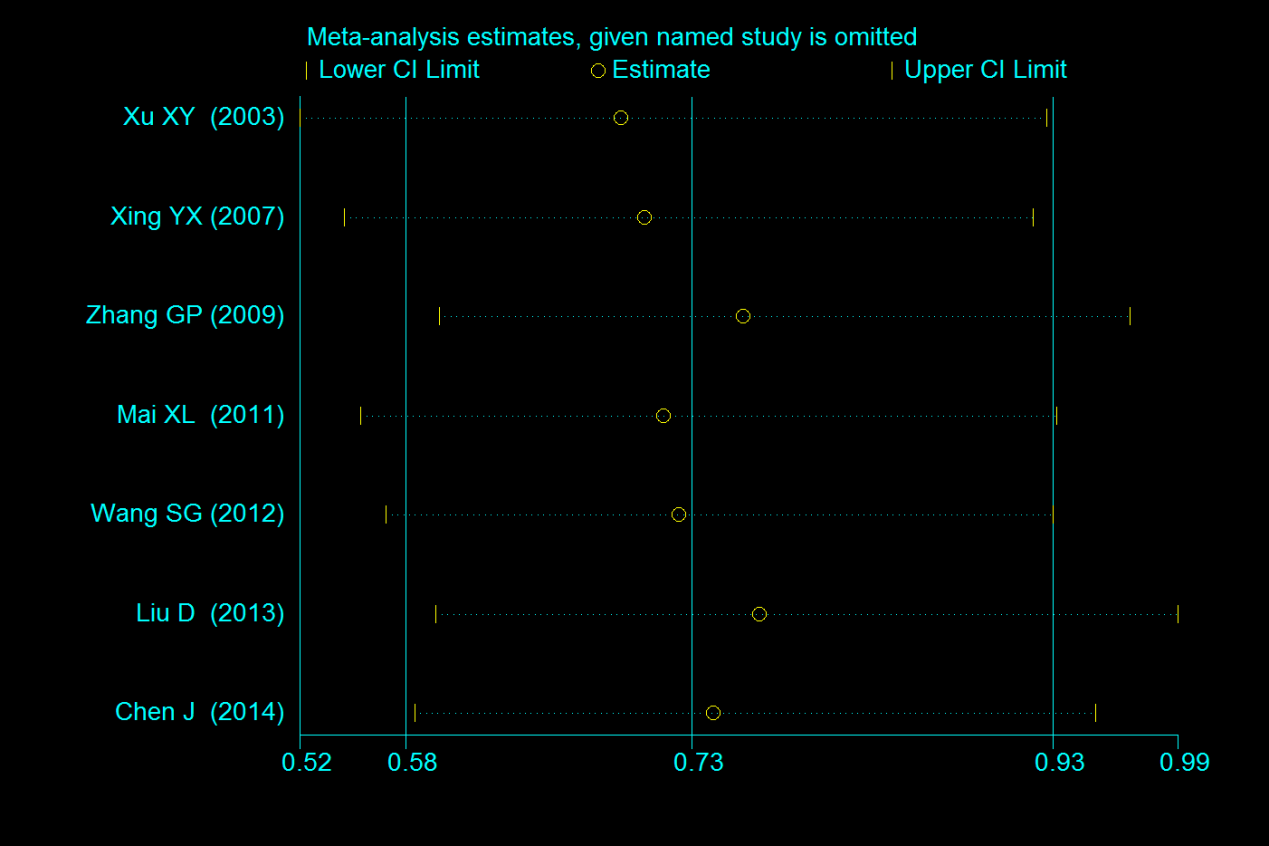


**S6 Fig. Sensitivity analysis of 7 studies with the fixed effects model for sexual pain within 6 months after delivery.**
